# Causes of embryo implantation failure: A systematic review and metaanalysis of procedures to increase embryo implantation potential

**DOI:** 10.3389/fendo.2024.1429193

**Published:** 2025-02-14

**Authors:** Francesco M. Bulletti, Romualdo Sciorio, Alessandro Conforti, Roberto De Luca, Carlo Bulletti, Antonio Palagiano, Marco Berrettini, Giulia Scaravelli, Roger A. Pierson

**Affiliations:** ^1^ Fertility Medicine and Gynecological Endocrinology Unit, Department Woman Mother Child, Lausanne University Hospital, Lausanne, Switzerland; ^2^ Department Neuroscience, Reproductive Science and Odontostomatology University of Naples Federico II, Naples, Italy; ^3^ Assisted Reproductive Technology (ART) Italian National Register, National Health Institute, Istituto Superiore di Sanità (ISS), Rome, Italy; ^4^ Department of Obstetrics, Gynecology and Reproductive Science, Yale University, New Haven, CT, United States; ^5^ Reproductive Science Pioneer, Assisted Fertilization Center (CFA), Naples, Italy; ^6^ Department of Statistical Sciences, University of Bologna, Bologna, Italy; ^7^ ART Italian National Register, National Health Institute, Istituto Superiore di Sanità (ISS), Rome, Italy; ^8^ Obstetrics and Gynecology, College of Medicine, University of Saskatchewan, Saskatoon, SK, Canada

**Keywords:** gestational carriers, clinical pregnancy outcomes, assisted reproductive technologies, infertility, implantation failure, euploid embryo transfer

## Abstract

**Introduction:**

Infertility is characterized by the failure to conceive after 12 months of unprotected sexual intercourse. In assisted reproduction technologies (ARTs), *in-vitro* fertilization and embryo transfer (IVF-ET) are pivotal, with the quality of embryo quality essential for successful implantation.

**Objective:**

This systematic review with meta-analysis aimed to explore the prevalence of embryonic factors involved in the implantation process, concentrating on the following research inquiries: 1) the implantation rates of euploid versus untested embryo transfers; 2) the efficiency of transferring good embryos in different age groups; 3) the impact of age on good embryo transfers to gestational carriers; and 4) the transfer of donated gametes/embryos. The goal is to identify critical points in implantation to improve therapies.

**Methods:**

A comprehensive literature search identified 1474 relevant papers, 11 of which met the inclusion criteria. The information was gathered using a standardized form, and the risk of bias was evaluated. A meta-analysis of subgroups to determine euploid embryo transfer efficiency was conducted to synthesize and explore the results. Furthermore, data extracted from registries document the persistent secondary role of extraembryonic determinants in successful implantation.

**Results:**

The meta-analysis demonstrated that preimplantation genetic testing for aneuploidy (PGT-A) significantly increased the odds of implantation. Age was found to influence extraembryonic factors, with older women experiencing reduced embryo implantation as gestational carriers. However, the overall incidence of extraembryonic factors was low. This review highlights the need to focus on PGT-A, diagnostic hysteroscopy, and endometrial receptivity for improving implantation rates.

**Conclusion:**

Implantation success in ARTs largely depends on embryo euploidy. While achieving three euploid embryos greatly increases success rates, it is challenging in older women. Extraembryonic factors, although present, have a marginal impact. Subsequent studies ought to concentrate on modulating endometrial responses immunologically and developing algorithms to improve the precision of predicting implantation success; as well as the timing of endometrial receptivity and the occurrence of dormant embryo phenomena also warrants further investigation.

## 1 Introduction

**Figure 1:**
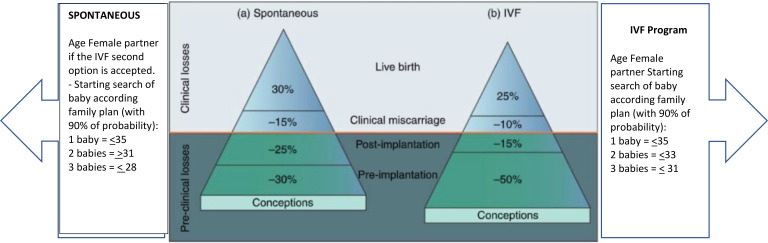
Picture depicts the estimation and prediction results for IVF cycles according to age and age. Modified with permission from (5).

**Figure 2:**
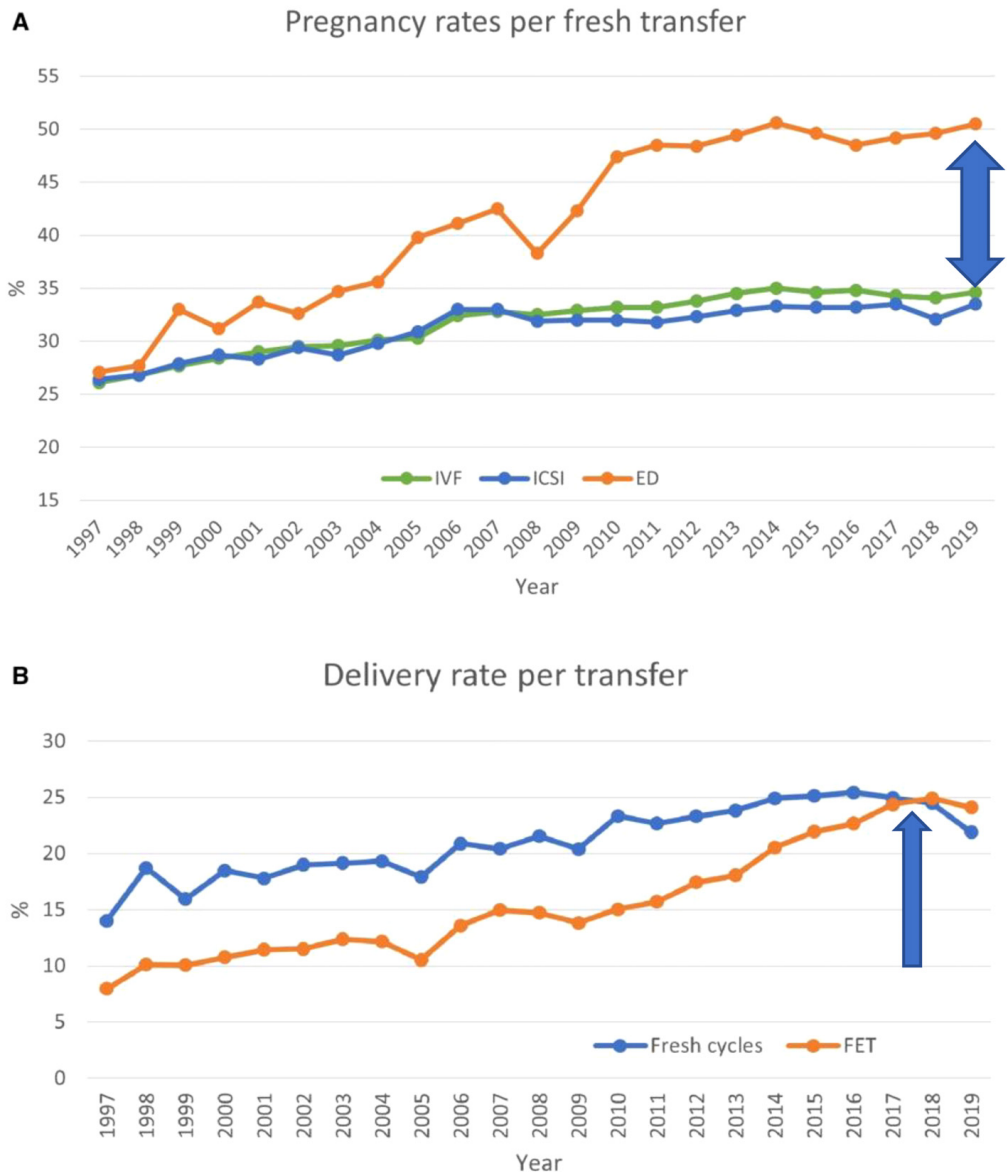
Pregnancy and delivery rates per transfer in Europe, 1997–2019. **(A)** Pregnancy rates for IVF versus ICSI and ED cycles. **(B)** Delivery rates for fresh versus frozen cycles. ED, egg donation. Modified from (1).

Infertility is the inability of the male or female reproductive system to achieve pregnancy after 12 months of unprotected intercourse, reduced to six months for women aged 35 or older (1–3). *In-vitro* fertilization and embryo transfer (IVF-ET), a key assisted reproductive technology (ART), has resulted in the birth of over 12 million children worldwide (1, 4). The critical stage in IVF-ET is embryo transfer and subsequent implantation into the maternal endometrium. However, implantation occurs in only 25% to 30% of transferred embryos, whether conceived *in vivo* or *in-vitro* (**Figures 1**, **2**). Embryo quality is the most significant feature affecting implantation (6–9). Achieving a 90% implantation success rate often requires at least three euploid embryos (8). Some studies estimate a 95% success rate with three consecutive euploid single embryo transfer (SET) in patients with optimal uterine conditions (9, 10). Younger women undergoing ART typically produce three euploid embryos with controlled ovarian stimulation (COS), but this becomes challenging for women over 37, which is the average age of IVF initiation in Europe (1). The rising age at which ART is sought underscores the need to better understand embryonic contributions to implantation success. Recent guidelines emphasize the importance of evidence-based investigations into implantation failure, avoiding unnecessary diagnostic and therapeutic procedures (6, 11). Our objective in this systematic review is to establish the incidence of embryonic factors in the implantation process with respect to the following questions:

- Implantation rates of euploid versus untested embryos.
- Efficiency of transferring high-quality non-biopsy embryos across age groups.
- Transfer success rates of good embryos into gestational carriers by age group.
- Outcomes of gamete/embryo donation programs involving donor embryos in women’s uteri. Identifying key factors in the implantation process will guide future research and improve therapeutic strategies.

## 2 Materials and methods

### 2.1 Protocol and registration

**Figure 3:**
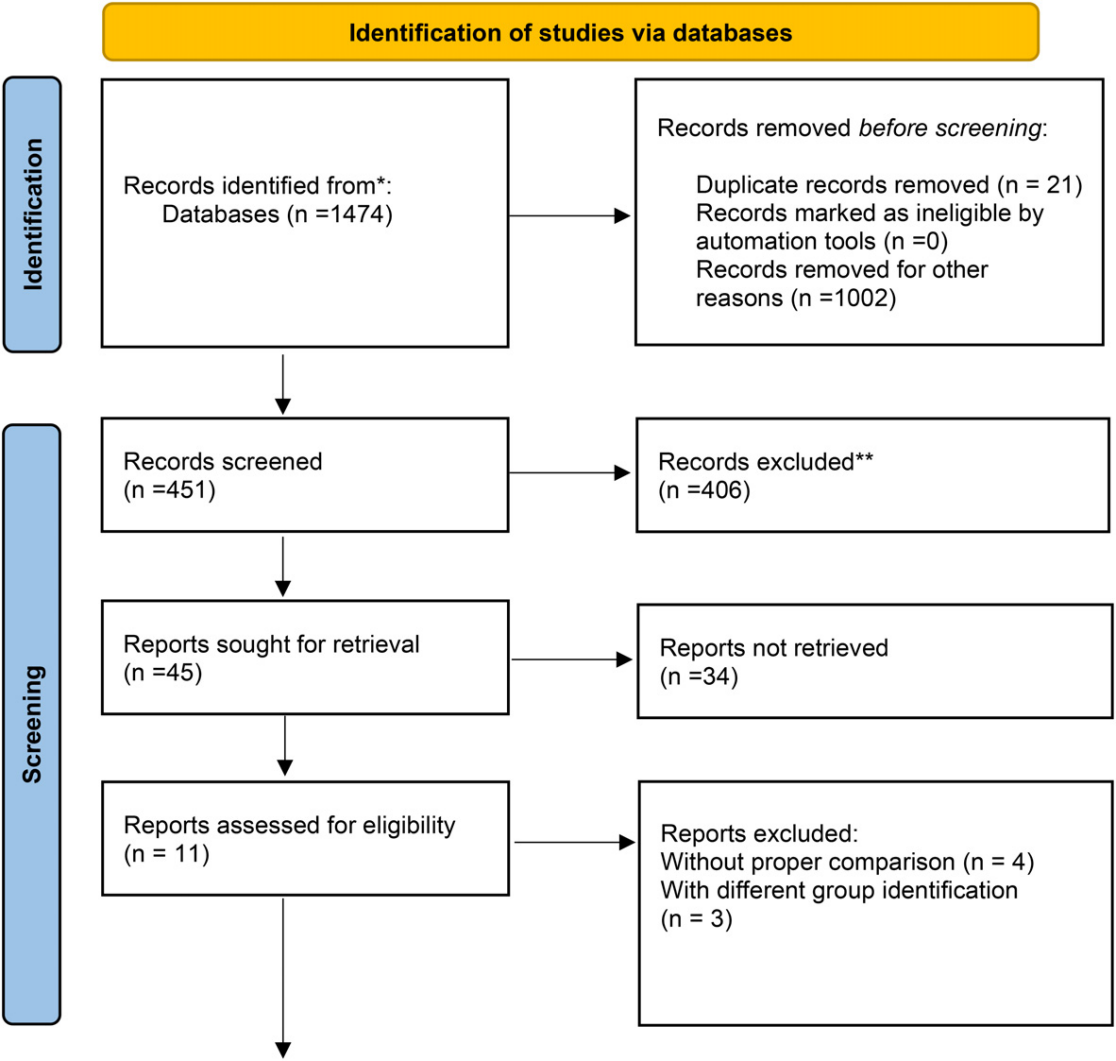
Data obtained from the databases were selected for quantitative assessment, as shown in the flow chart. The qualitative assessments reported here were obtained from registry data and were not included in the meta-analysis. * From (12).

This systematic review and meta-analysis were conducted by searching electronic databases, including MEDLINE, Web of Science, and Scopus, covering the period from January 1980 to December 2023 (**Figure 3**). The review adhered to the Preferred Reporting Items for Systematic Reviews and Meta-Analyses (PRISMA) guidelines (13) and was registered with INPLASY202410008 (DOI: 10.37766/inplasy2024.1.0008). The Rayyan framework, an AI-powered tool, was used for article screening. Additionally, manual searches of reference lists from included studies complemented the electronic database search. Exclusion criteria included non-English reports, animal studies, and research predating 1980.

## 3 Information sources

### 3.1 Database search

The search strategy employed a comprehensive combination of keywords related to assisted reproductive technologies (ARTs), embryo implantation, and related topics. Examples of search terms included:

- “*in-vitro* fertilization,”
- “assisted reproductive techniques,”
- “embryo implantation,”
- “endometrial receptivity,”
- “preimplantation genetic testing (PGT-A),” and
- “gestational carrier.”

The search was restricted to English-language articles to ensure consistency in data extraction and interpretation.

### 3.2 Registry Data

Data were collected from multiple ART registers, including:

- USA: SART and CDC data for gestational carrier (GC) and non-GC cycles (2019–2020).
- Australia/New Zealand: ANZARD data (2020).
- Portugal: CNPMA data.
- UK: HFEA data (2014–2016).

These registries provided detailed information on ART outcomes, including embryo transfers, pregnancy rates, and delivery rates.

### 3.3 Study Selection

A rigorous multi-step process was applied to select studies for inclusion:

**Inclusion criteria**

- Studies published in English.
- Original research articles, clinical trials, observational studies, and registry data.
- Studies investigating the role of embryonic factors in human embryo implantation.
- Studies reporting outcomes related to embryo quality, age, number of previous implantation failures, uterine factors, endometrial thickness, and other factors influencing successful implantation.

**Exclusion criteria**

- Studies with insufficient data or inadequate reporting.
- Non-original articles (e.g., reviews, case reports, editorials).
- Studies not directly related to the research question.

Two independent reviewers screened titles and abstracts to identify relevant studies, followed by a full-text review. Discrepancies were resolved through discussion and consensus.

Despite an initial pool of 1,474 articles, most were excluded due to insufficient data, lack of relevant outcomes, or failure to meet inclusion criteria. Only 11 studies remained after the rigorous screening process (**Figure 3**). This small number reflects the stringent selection process and the specificity of the research focus, which required high-quality data directly addressing the impact of embryonic and extraembryonic factors on implantation.

### 3.4 Data extraction and quality assessment

A standardized data extraction form was used to collect study details, patient demographics, interventions, and outcomes. The Cochrane Risk of Bias tool (randomized trials) and Newcastle–Ottawa Scale (observational studies) were applied to assess study quality.

## 4 Data synthesis and analysis

### 4.1 Meta-analysis

Data were synthesized using random-effects or fixed-effects models, depending on heterogeneity assessed via the I² statistic. Pooled effect estimates and 95% confidence intervals (CI) were calculated for categorical outcomes using odds ratios (ORs). Analyses were performed using R version 4.3.1 with the meta package (14, 15).

## 5 Subgroup and sensitivity analyses

### 5.1 Subgroup analysis

Explored heterogeneity sources such as study design, patient characteristics, or methodological differences.

### 5.2 Sensitivity analysis

Tested the robustness of findings by excluding high-risk studies or varying specific characteristics.

#### 5.2.1 Publication bias

Publication bias was evaluated using funnel plots and statistical tests like Egger’s test, where applicable.

#### 5.2.2 Reporting

Findings were reported in accordance with PRISMA guidelines (13) to ensure transparency and reproducibility.

## 6 Results

### 6.1 Study selection

The initial literature search yielded 1,474 studies addressing the research questions. After three rounds of screening, 11 studies were selected for final analysis (**Table 1**). Four studies were excluded due to the lack of comparable data across study arms.

**Table 1:** The number of studies selected for the meta-analysis that focused on the implantation rate of embryos with respect to euploid versus untested embryos was 4.

Reference	Study design	Population	Group A (intervention)	Group B (comparison treatment)	Group C (no treatment)	Intervention	Comparison	no treatment/ placebo	Implantation rate (%) A	Implantation rate (%) B	Implantation rate (%) C	MEAN DIFF_IR	CI IR	P VALUE IR	LBR (%) A	LBR (%) B	LBR (%) C	MEAN DIFF LBR
(16)	RCT	RIF patients	72		67	PGS before blastocyst transfer		Blastocyst transfer without PGS	21,4		25,3			NS				
(17)	Observational study	RIF or RM patients undergoing PGD-A	69	69		Vitrified/warmed oocytes (first ovarian stimulation)	Fresh oocytes (second ovarian stimulation)		56	60,9				0,05				
(18)	Retrospective cohort study	RIF patients	14		14	PGT-A before embryo transfer		Embryo transfer without PGT-A	90		76,9			0,6036	90		69,2	
(19)	Retrospective cohort study	RIF patients	30		42	PGT-A before embryo transfer		Embryo transfer without PGT-A	69,5		33,3	0,218	0.073_0.654	0,005	47,8		19	0,256
(20)	RCT	RIF patients	48		43	PGS before blastocyst transfer		Blastocyst transfer without PGS	36,6		21,4			NS	47,9		27,9	
(21)	Prospective pilot study	RIF patients	42		50	PGT-A before embryo transfer		Embryo transfer without PGT-A							62,5		31,7	3,75
(22)	Retrospective cohort study	Patients with endometriosis undergoing fresh ET	52		287	PGT-A before embryo transfer		Embryo transfer without PGT-A	45,2		49,7							
(23)	Retrospective cohort study	RIF patients	64		94	Noninvasive chromosome screening (NICS) before blastocyst transfer		Blastocyst transfer without NICS							29,7		21,3	1,96
(24)	Prospective study	RIF patients	54		86	PGS before embryo transfer		Embryo transfer without PGS							14,8		24,41	-9,6
(25)	Clinical study	RIF and good prognosis patients	43	33	45	RIF patients PGS before embryo transfer	RIF patients without PGS before embryo transfer	Good prognosis patients without PGS before embryo transfer	68,3	22	70,5			<0.001				
(26)	Retrospective cohort study	Moderate RIF patients	144		1840	PGT-A before embryo transfer		Embryo transfer without PGT-A	45,9		35,9	1,34	1.17_1.55	<0.001				

### 6.2 Delivery rates and implantation outcomes

**Figure 4:**
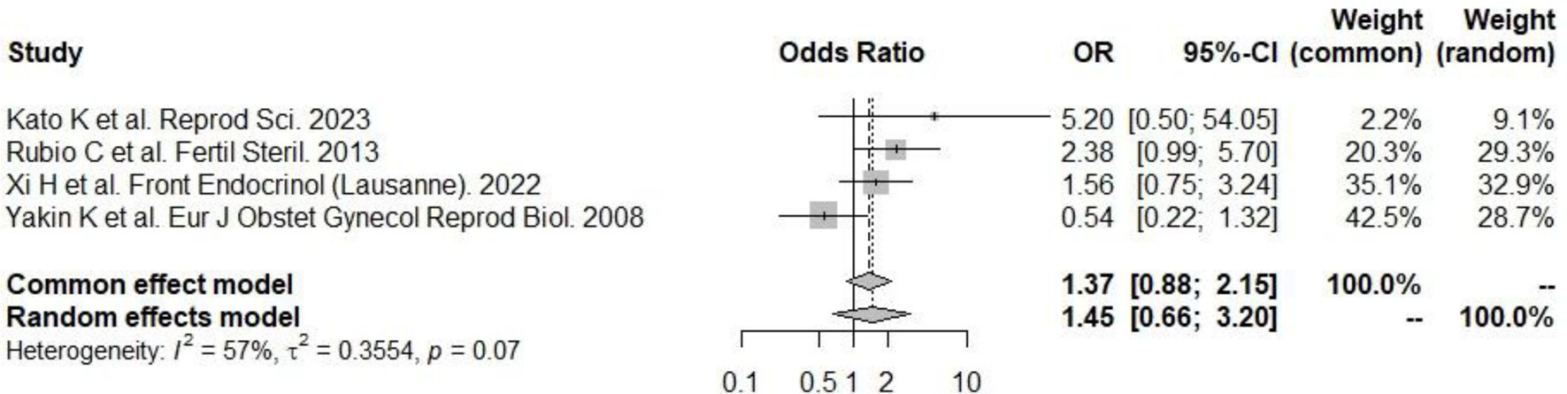
PGT-A and live birth rate. There was a significant positive effect on the odds of implantation: according to the random effects model (to be preferred, given the presence of heterogeneity), the odds of implantation were significantly greater in the PGT-A group.

Recent European data for fresh and frozen embryo transfers in IVF cycles (2017–2018) reported delivery rates below 25% (**Figures 2**, **4**) (1). Embryo donation results revealed pregnancy and delivery rates of 45.1%, 43.4%, and 41.9% for age groups <34, 35–39, and >40 years, respectively. Corresponding live birth rates (LBR) were 31.4%, 33.8%, and 29.1% (**Table 2**). These findings suggest that while extraembryonic factors play a role, they are secondary to embryo quality in determining implantation success.

**Table 2:** Transfer, pregnancy, and delivery rates by age distribution (years) of women treated with the ED in 2019.

Country	Transfers (%)			Pregnancy rates (%)			Delivery rates (%)		
	<34	35-39	≥40	<34	35-39	≥40	<34	35-39	≥40
Albania	20	0	80	50		62,5	50		37,5
Armemia	19,9	36	44,1	63	45,4	38,8	59,3	38,3	28,8
Austria									
Belarus	17,4	34,1	48,6	41,7	42,6	44,8	12,5	31,9	17,9
Belgium	21,7	21,7	56,6	33,3	29,8	26,7	24,7	23,3	18,9
Bosnia-Herzegovina, Federation part									
Bulgaria									
Czech Republic	11,2	21,4	67,4	45,5	44,9	42,3	24,3	24	21,5
Denmark	17,2	25,2	57,6	35,5	25,6	25,2	20,8	14,2	13,5
Estonia	6,1	15,9	78	40	46,2	38	26,7	30,8	30,7
Finland	15,4	20,6	64,1	34,9	33,1	33,5	21,7	24,6	23,8
France	36	42	21,9	30,1	26,4	31,1	24,9	22,1	26,6
Germany									
Greece	5,2	11,8	83	60,6	70,7	51,7	30,3	48,7	34,7
Hungary									
Iceland	23,6	16	60,4	32	29,4	42,2	28	29,4	32,8
Ireland	25	0	75	50		33,3	50		16,7
Italy	5	15	80	43,6	37,4	37,1	25,3	26	25,4
Kazakhstan	24,6	27,7	47,8	54,8	54,8	46,9	42,6	40,9	33,4
Latvia	9,7	12,3	78,1	53,3	36,8	44,6	46,7	26,3	24,8
Lithuania	50	0	50	100		100	100		100
Luxembourg									
Macedonia	4,7	15,6	79,7	40	39,4	33,7	40	30,3	17,8
Malta									
Moldova									
Montenegro									
Norway									
Poland	16,8	30,6	52,7	51,4	42,2	38,4	33,3	31,4	24,7
Portugal	6	17	77	43,1	47,1	44,8	31,2	38	34,3
Russia	17,8	27,1	55,1	52,3	52,3	42,1	38,2	39,8	28,2
Serbia									
Slovakia									
Slovenia	0	0	100						
Spain	5,5	19,2	75,3	48,6	50,4	47,1	37	38,6	34,5
Sweden	48,3	34,4	17,2	38,1	39,5	31,6	31,3	31,6	15,8
Switzerland									
The Nederlands									
Turkey									
Ukraine	23,7	30,9	45,4	67,9	65,5	61,1	59,5	62,6	50,5
UK	18,8	21,6	59,6	0	0				
All*	**11,4**	**21,2**	**67,5**	**43,4**	**45,1**	**41,9**	**31,4**	**33,8**	**29,1**

*All: percentage of cycles per age group is computed among all countries giving the distribution. Pregnancy and delivery rates are computed for the countries providing them.

### 6.3 Gestational carrier outcomes

Reproductive outcomes in gestational carriers (GCs) were significantly higher than those in non-gestational carriers (noGCs). For fresh nondonor oocytes, the implantation rate (IR) was 14.5% higher in GCs than in noGCs, and for fresh donor oocytes too, the IR was 11% higher in GCs (**Table 3**). When the reproductive outcomes of GCs treated with fresh or donor oocytes were compared to those of noGCs, the implantation rate was greater for fresh nondonor GCs than for fresh nondonor oocytes that were not helped from GC, and when fresh donor oocytes were used, the IR of GCs was greater than that of noGCs (**Table 3**). All the data emphasize that there is a significant, albeit secondary, role of extraembryonic factors in the success of implantation. Additionally, data from the IVF Australia registry and other studies confirm the enhanced implantation and live birth rates (LBR) in GCs compared to noGCs (**Table 4**). Namath and collaborators (27) reported that LBR for single embryo transfer (SET) in GCs was 36.8%, significantly lower than the 51.3% reported for double embryo transfer (DET) (p < 0.001). However, there was no significant difference between LBRs with preimplantation genetic testing for aneuploidy (PGT-A) and without PGT-A (36.8% *vs*. 36.7%). These findings suggest a limited role of PGT-A in improving outcomes within this cohort. Nevertheless, prior full-term delivery and rigorous GC screening significantly enhance the likelihood of uncomplicated pregnancies and healthier outcomes (28).

**Table 3:** Register of assisted reproductive technology in Australia and New Zealand 2020.

Outcomes of surrogate gestational carrier cycles, Australia and New Zealand, 2020.		
*Gestational carrier*	*No Gestational Carrier*	
• Clinical pregnancies per embryo transfer (%) 44.2 • Live Birth rate per embryo transfer (%) 39.1	**FET** • Clinical pregnancies per embryo transfer (%) 38.7 • Live Birth rate per embryo transfer (%) 31.3	**Fresh** • Clinical pregnancies per embryo transfer (%) 32.6 • Live Birth rate per embryo transfer (%) 25.3

The percentage of live births per embryo transfer in gestational carriers seems to be greater than that in non-gestational carriers (Report 2020 of ANZARD https://npesu.unsw.edu.au/surveillance-reports).

**Table 4A:** The use of non-donor oocytes versus donor oocytes in non-gestational carrier versus gestational carriers indicates a moderate role of uterine factors as implantation determinants.

Reproductive outcomes for gestational carrier and non-gestational carrier cycles using fresh nondonor or fresh donor oocytes, United States, 2009–2013.						
Fresh no donor oocytes						
	Gestational Carrier		Non Gestational Carrier			
Variable	N	%	N	%	RR(95% CI)	aRR(95% CI)
Among Transfers						
Implantation Rate	2,462	30.3	224,974	25.9	1.17(1.11-1.22)	1.22(1.17-1.26)
Clinical Pregnancy	1,918	51.8	178,557	44.7	1.16(1.12-1.20)	1.14(1.10-1.19)
Live Birth	1,537	41.5	145,963	36.5	1.14(1.09-1.18)	1.17(1.12-1.21)
Fresh donor oocytes						
Among Transfers						
Implantation Rate	3,825	**53.3**	38,45	**47.4**	1.12(1.07-1.18)	1.11(1.07-1.15)
Clinical Pregnancy	2,669	69.7	28,898	65.0	1.07(1.04-1.10)	1.05(1.03-1.08)
Live Birth	2,32	60.5	24,537	55.2	1.10(1.06-1.13)	1.08 (1.05-1.11)

Adapted with permission form 28.

**Table 4B:** Live birth rates for euploid embryo transfer by age group and uterine environment.

Age Group	Own Embryos (%)	Gestational Carrier (%)
<35	65	72
35–37	55	68
38–40	42	60
41–42	32	50
>42	15	40

Table compares outcomes for women using their own oocytes with those involving a gestational carrier. (Sources: SART registry, https://www.sart.org/; HFEA registry, https://www.hfea.gov.uk/about-us/data-research/; Australian and New Zealand Assisted Reproduction Database (ANZARD), https://www.unsw.edu.au/research/npesu/clinical-registries/anz-assisted-reproduction-database#:~:text=The%20Australia%20and%20New%20Zealand,and%20New%20Zealan%20fertility%20clinics).

**Table 4C:** Live Birth rates (LBR) for euploid embryo transfers by attempt number and embryo type.

Transfer Attempt	Own Embryos (%)	Gestational Carrier (%)
1st Transfer	60	70
2nd Transfer	50	65
3rd Transfer	40	60
4th Transfer	35	55
5th Transfer	30	50

This table displays live birth rates segmented by the number of transfer attempts and whether the embryo was transferred into the patient's own uterus or into a gestational carrier. The data reveal a general trend of decreasing success rates with each successive transfer attempt for both groups. However, live birth rates consistently remain higher when using a gestational carrier, particularly in later attempts. (Sources: SART registry, https://www.sart.org/; HFEA registry, https://www.hfea.gov.uk/about-us/data-research/; Australian and New Zealand Assisted Reproduction Database (ANZARD), https://www.unsw.edu.au/research/npesu/clinical-registries/anz-assisted-reproduction-database#:~:text=The%20Australia%20and%20New%20Zealand,and%20New%20Zealan%20fertility%20clinics).

**Table 4D:** Pregnancy rates per attempt for uterine factor infertility cases.

Transfer Attempt	Own Uterus (%)	Gestational Carrier (%)
1st Attempt	45	70
2nd Attempt	35	65
3rd Attempt	28	60
4th Attempt	20	55
5th Attempt	15	50

This table presents hypothetical pregnancy rates per attempt for patients with uterine factor infertility, comparing outcomes for those using their own uterus versus a gestational carrier. The data indicate that pregnancy rates are significantly higher for each attempt when a gestational carrier is used, as opposed to the patient’s own uterus. A 2020 CDC report on ART outcomes further supports this trend, showing that gestational carrier cycles have high success rates, particularly when using donor or euploid embryos (44, 45).

### 6.4 Importance of embryo quality

Franasiak and colleagues (29) demonstrated that achieving a 95% sustained implantation rate requires three consecutive euploid single embryo transfers (SET) (**Tables 5**, **6**), emphasizing the critical role of embryo quality. Data from the SART registries (2019–2020) compared reproductive outcomes in patients undergoing IVF with their own eggs and PGT-A versus patients using GCs. The first embryo transfer of oocytes with PGT-A showed dramatically better outcomes for GCs compared to noGCs, with a smaller difference observed in second or later transfers. This highlights the role of extrauterine factors, particularly in the early stages of embryo transfer (**Tables 7A**-**C**).

**Table 5:** Estimation model for the number of unscreened good-quality embryos needed to be equivalent to 3 successive euploid embryo transfers and achieve a 95% chance of sustained implantation on the basis of the observed aneuploidy rate. Adapted from 30.

Age (y)	Observed aneuploidy rate	No. of untested blastocysts to achieve a 95% chance of sustained implantation
<35	20%	4
35–37	30%	5
38–40	50%	7
41–42	70%	13
≥43	85%	27

**Table 6:** Sustained implantation rate after embryo transfer with or without preimplantation genetic testing for aneuploidy by age in the 2020 Society for Reproductive Technology national outcomes report.

Type of ART cycle	<35y	35–37y	38–40y	41–42 y	≥43y
**PGT-A cycles**	62.5%	60.8%	58.7%	53.7%	48.3%
**Non-PGT-A cycles**	46.8%	41.1%	34.7%	25.5%	16.7%

The well-known dramatic reduction observed in non-PGT-A cycles was not observed in the PGT-A cycles, but an approximately 23% reduction was still observed between the implantation rates of euploid embryo transfers in the <35 years old group and those in the >43 years old group. Thus, the influence of extraembryonic factors on successful implantation is emphasized.

**Table 7A:** The SART registries 2019 and 2020, which reported the reproductive outcomes of patients who underwent IVF with their own eggs and PGT-A without using GCs at the first embryo transfer.

PATIENT'S OWN EGGS					
LIVE BIRTHS PER INTENDED EGG RETRIEVAL (FIRST EMBRYO TRANSFERS)					
NO GESTATIONAL CARRIER WITH PGT					
**2020**	<35	35-37	38-40	41-42	>42
Number of cycle started	19.905	14.868	14.024	6.311	2.960
Singleton Births x cycle, %	43,9	37,2	28,5	17,3	7,7
Live births x cycle, %	45,5	38,4	29,3	17,6	7,8
Confidence Intervals	44,8-46,2	37,6-39,2	28,5-30,1	16,6-18,5	6,8-8,8

A significant decrease in performance with increasing age is a sign of an increase in the incidence of extraembryonic factors associated with successful implantation.

**Table 7B:** The number of live births per intended egg retrieval (second or greater number of embryo transfers) was determined by using one’s own eggs with PGT without a gestational carrier.

PATIENT'S OWN EGGS					
LIVE BIRTHS PER SECOND OR LATER EMBRYO TRANSFERS					
NO GESTATIONAL CARRIER WITH PGT					
**2020**	<35	35-37	38-40	41-42	>42
Number of thaw procedure	8.992	6.765	5.520	2.017	1.194
Singleton Births x cycle, %	48,6	49,9	49,0	48,1	45,6
Live births x cycle, %	50,9	51,7	50,5	49,5	46,5
Confidence Intervals	49,8-51,9	50,5-52,9	49,2-51,8	47,3-51,7	43,7-49,3

There were no significant changes in the implantation rate among the age groups, thus indicating that a selection group where possible previously detected extraembryonic factors were excluded or treated.

**Table 7C:** Live births per intended egg retrieval (first embryo transfers) by using one’s own eggs with PGT with a gestational carrier.

PATIENT'S OWN EGGS					
LIVE BIRTHS PER INTENDED EGG RETRIEVAL (FIRST EMBRYO TRANSFERS)					
GESTATIONAL CARRIER WITH PGT					
2020	<35	35-37	38-40	41-42	>42
Number of cycle started	337	193	154	51	11
Singleton Births x cycle, %	60,8	60,1	55,2	45,1	4/11
Live births x cycle, %	62,6	62,7	59,1	47,1	6/11
Confidence Intervals	57,4-67,8	55,9-69,5	51,3-66,9	33,4-60,8	

The results indicate a nonsignificant difference in implantation rate with age, as indicated by the absence of extraembryonic factors in gestational carriers (those who already had children).

### 6.5 Extraembryonic contributions

The influence of extrauterine factors was particularly evident in first embryo transfers performed via GCs, with reduced impact observed in second or later transfers. This highlights the supportive role of extraembryonic environments in promoting implantation and ongoing pregnancies. However, when analyzing extraembryonic factors based on studies that provide evidence or, in some cases, lack definitive evidence but are supported by suggestive findings that warrant further exploration through randomized controlled trials (RCTs). Conversely, other factors neither show supporting evidence nor suggestions from studies that are free from significant risk of bias. Careful screening of GCs and use of high-quality embryos can maximize the likelihood of safe and successful pregnancies. Recent research highlights the dominant role of embryonic factors in successful implantation, particularly in studies involving euploid embryo transfers, oocyte donor programs, and PGT-A-screened single embryo transfers (SET). Evidence consistently confirms the superiority of embryonic factors over extraembryonic factors (9). Implantation rates for euploid embryo transfers approach 95% after three sequential SETs, underscoring the pivotal role of embryo quality (9). However, data from gestational carrier (GC) studies reveal a more nuanced picture of extraembryonic factors, which remain secondary but significant. For instance, SET using a GC consistently shows better outcomes than transfers to the biological mother’s uterus, even with PGT-A screened embryos. The implantation rate (IR) of fresh donor oocytes in GCs is approximately 11% higher than in non-gestational carriers (noGCs) (**Tables 7C**, **D**). Age, however, emerges as a key modulating factor. Older recipients exhibit lower implantation rates even in GC scenarios, indicating an age-related influence on extraembryonic factors such as uterine environment and endometrial receptivity (31).

**Table 7D:** The number of live births per intended egg retrieval (those who underwent two or more embryo transfers) determined by the use of their own eggs with PGT with a gestational carrier.

PATIENT'S OWN EGGS					
LIVE BIRTHS PER SECOND OR LATER EMBRYO TRANSFERS					
GESTATIONAL CARRIER WITH PGT					
**2020**	<35	35-37	38-40	41-42	>42
Number of thaw procedure	385	373	364	180	190
Singleton Births x cycle, %	50,4	56,3	45,3	49,4	48,4
Live births x cycle, %	53,5	59,2	48,9	51,7	49,5
Confidence Intervals	48,5-58,5	54,3-64,2	4,8-54	44,4-59	42,4-56,6

The performances indicate a nonsignificant difference in the implantation rate with age, which is a sign of the absence of extraembryonic factors in gestational carriers (those who already had children).

### 6.6 Age-related extraembryonic

**Figure 5:**
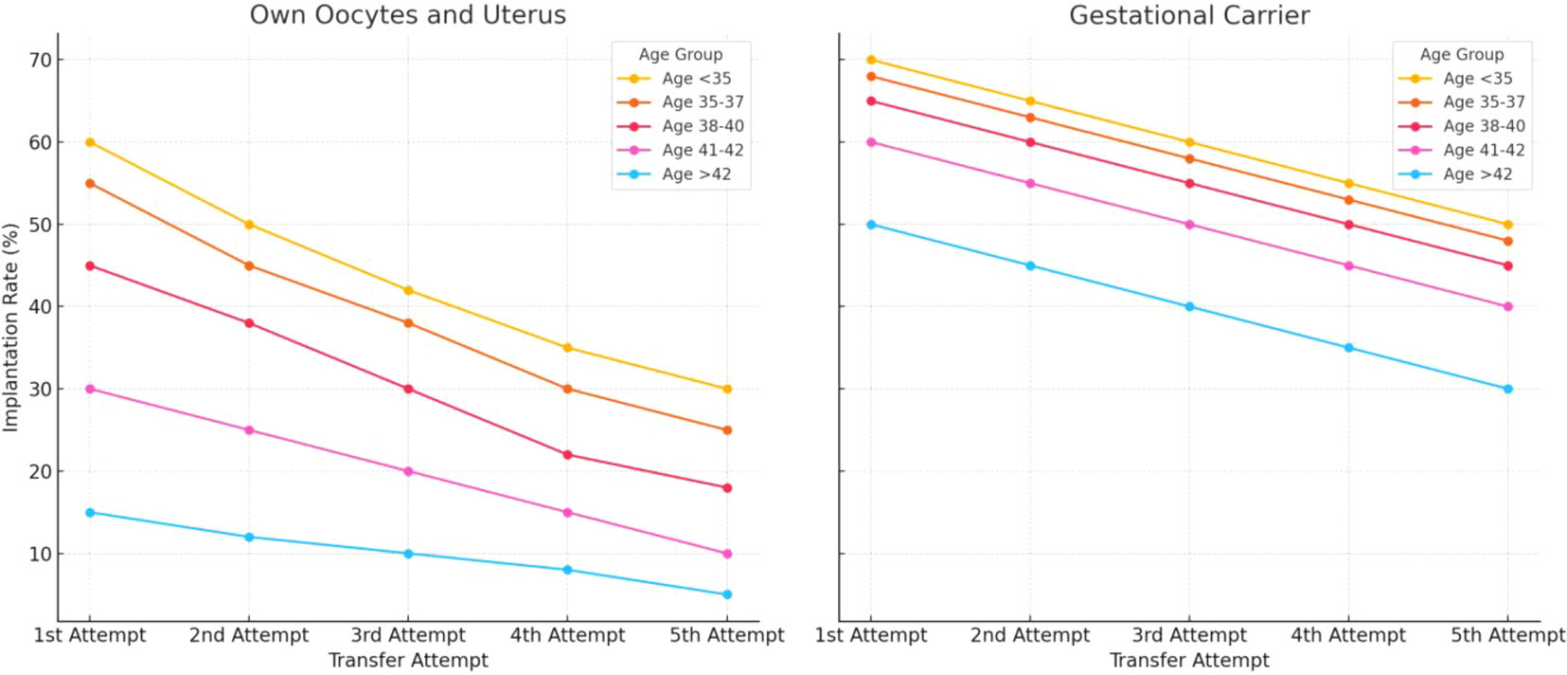
Estimated implantation rates across different age groups, comparing outcomes for women using their own uterus and eggs versus those using a gestational carrier. Data trends are derived from annual reports by the Society for Assisted Reproductive Technology (SART) and the Centers for Disease Control and Prevention (CDC) in the United States. These reports provide detailed statistics on ART (Assisted Reproductive Technology) outcomes, including implantation rates, use of gestational carriers, and related metrics. The data is sourced from SART’s Clinic Outcome Reporting System, which collects ART cycle information from U.S. clinics to offer insights into IVF cycles, pregnancy and implantation rates, live birth rates, and more, categorized by patient age and cycle type. (Source: SART registry, https://www.sart.org/).

As women age, their uterine environment may become less supportive of pregnancy due to age-related declines in endometrial receptivity and vascular health (31–37). Older patients often benefit from gestational carriers, who are typically younger and have healthier uterine conditions. Influences Age significantly impacts uterine factors such as endometrial thickness, uterine pathologies (e.g., myomas, polyps, and adhesions), and hormonal regulation (38). For patients using their own uterus and oocytes, implantation rates generally decrease with age and with each successive transfer attempt. Younger age groups (<35 and 35–37) initially show higher implantation rates but still experience declines with multiple attempts (**Figure 5**). This influence persists even in GCs, emphasizing the need to account for uterine aging in assessing implantation outcomes. Age-related epigenetic dysfunction of the endometrium, including altered receptivity and decidualization processes, has been proposed as a key factor in implantation failures (4, 39). While embryo quality remains the primary determinant of implantation success, the *“endometrium as a biosensor”* hypothesis posits that a non-viable embryo will not be accepted by the uterine environment, irrespective of other factors (40). This underscores the interplay between embryonic and extraembryonic factors during implantation.

### 6.7 Role of gestational carriers in addressing RIF

Patients with uterine factor infertility, or uterine anomalies and adhesions, might represent an ideal candidate for using a gestational carrier (GC). This approach is especially beneficial for women who have experienced implantation failures, or multiple failed embryo transfers despite using high-quality, euploid embryos. Clinical evidence indicate that these patients benefit significantly from a GC, as the carrier’s healthy uterine environment can improve embryo implantation and increase live birth rates (41–43). Studies have shown that live birth rates for patients with uterine factor infertility using gestational carriers often approach those of women without uterine issues using their own embryos (**Tables 4B**–**D**). Data from SART, UK, and Australian fertility registries show that euploid embryo transfer success rates vary based on the woman’s age and the use of a gestational carrier (41–43). Additional studies comparing fresh and frozen embryo transfers in GCs demonstrate improved outcomes compared to noGCs, highlighting the importance of uterine receptivity (27). Nevertheless, age-related uterine changes remain evident even in GCs, as seen in declining implantation rates for older age classes (**Tables 7A**, **B**). Interestingly, PGT-A does not significantly enhance outcomes in GCs, with comparable live birth rates (LBRs) observed for PGT-A (36.8%) and non-PGT-A (36.7%) embryos (27).

### 6.8 Clinical implications

Although embryonic factors dominate implantation outcomes, addressing age-related uterine changes and optimizing extraembryonic conditions are essential for improving ART success rates. Diagnostic and therapeutic efforts should focus on:

- Comprehensive uterine evaluation (e.g., hysteroscopy, 3D ultrasound).
- Addressing age-related uterine factors, such as thin endometrium (<7 mm), hydrosalpinx, and endometritis.
- Incorporating targeted interventions (e.g., antibiotics for chronic endometritis, hormonal therapies) based on patient-specific risk factors (46).

In conclusion, while the role of extraembryonic factors is secondary, age-related uterine influences must be accounted for in ART protocols, particularly in older women and GC programs. Combining high-quality euploid embryos with optimal uterine conditions provides the best chance for successful implantation and live birth.

## 7 Discussion

### 7.1 Future research directions

Embryonic implantation remains a complex, multifactorial process influenced by both embryonic and extraembryonic factors. While advances such as preimplantation genetic testing for aneuploidy (PGT-A) have improved embryo selection, significant gaps persist in understanding and addressing extraembryonic factors, particularly those related to immunomodulation and personalized approaches to optimize implantation success. Below, we outline key areas for future research:

### 7.2 Immunomodulation in endometrial receptivity

The endometrial immune response plays a critical role in the embryo’s attachment and subsequent implantation. Recent studies suggest that the maternal immune system acts as a modulator, balancing tolerance to the semi-allogenic embryo while preserving the ability to identify and reject non-viable embryos (40). However, the precise mechanisms of this immunological balance remain poorly understood.

Future studies should focus on:

- *Decoding Immunological Pathways*: Investigating the role of uterine natural killer (uNK) cells, macrophages, and T-regulatory cells in supporting implantation. Aberrant immune responses, such as an overactive Th1/Th2 balance, have been linked to implantation failure (46).
- *Targeted Immunotherapies:* Developing personalized immunomodulatory therapies, such as vitamin D supplementation, tacrolimus, or low-dose aspirin, which have shown promise in addressing chronic endometritis and immune-mediated recurrent implantation failure (RIF) (6, 46).
- *Molecular Diagnostics:* Advancing diagnostic techniques to identify immune-related implantation barriers. For example, profiling cytokine levels and identifying biomarkers of inflammation could provide actionable insights for treatment.

By focusing on the immunological aspects of implantation, clinicians may better address unexplained RIF and enhance pregnancy outcomes in women undergoing ART.

### 7.3 Personalized algorithms for implantation success

The multifactorial nature of implantation requires an integrative approach to treatment. Emerging computational tools, such as machine learning algorithms, have the potential to revolutionize ART by integrating diverse patient data and providing tailored recommendations.

Future research should aim to:

- *Develop Dynamic Predictive Models:* Incorporating data on embryo quality (e.g., PGT-A results), endometrial receptivity, patient demographics, and hormonal profiles into advanced algorithms. These models can predict the optimal timing for embryo transfer and identify high-risk patients.
- *Leverage Big Data:* Building shared, global databases that aggregate information from ART centers worldwide. Such databases could track temporal coordination between embryonic and endometrial development, uncovering patterns that improve clinical decision-making.
- *Incorporate Real-Time Adjustments*: Utilizing real-time data (e.g., ultrasound findings, hormonal levels) to refine treatment protocols dynamically. This approach could significantly improve outcomes, particularly in patients with a history of implantation failure.

One promising avenue involves time-lapse imaging to monitor embryonic development alongside endometrial receptivity. This technology may help identify subtle deviations in the implantation window or embryonic dormancy: a phenomenon observed in other species, with potential relevance to human ART (31).

### 7.4 Advancing the concept of the “Endometrial Biosensor”

The idea that the endometrium functions as a biosensor, selectively supporting viable embryos, provides a compelling framework for future research (40). This concept underscores the importance of understanding the molecular cross-talk between the embryo and the endometrium.

Key research directions include:

- *Endometrial Biomarkers:* Identifying molecular signals, such as cytokines or exosomal markers, that indicate endometrial receptivity.
- *Epigenetic Profiling:* Exploring age-related epigenetic changes in the endometrium to predict implantation potential and develop interventions to reverse senescence-related receptivity loss (39).
- *Exploring Dormant Embryo Phenomena:* Investigating whether human embryos, like those in other species, can delay development until the endometrium becomes receptive.

### 7.5 Clinical trials for RIF interventions

Given the heterogeneity of factors contributing to RIF, future research should prioritize well-designed, multi-center clinical trials that evaluate the effectiveness of combined diagnostic and therapeutic strategies. For example, the OPTIMUM trial demonstrated improved outcomes in patients treated for chronic endometritis and immune-related issues, yet further studies are needed to validate these findings across diverse populations (46).

### 7.6 Time-lapse studies on endometrial receptivity

The precise timing of the endometrial implantation window is critical. Research should further explore the molecular and structural changes that define this window, particularly in older women or those with endometrial pathologies. Identifying the optimal interval for transfer may mitigate the impact of age-related uterine changes on implantation success.

## 8 Conclusion and research priorities

While the role of embryonic factors in implantation is well-established, addressing the immunological and extraembryonic factors remains essential for improving ART outcomes. The following priorities should guide future research:

- Expand immunomodulatory strategies to address maternal immune dysregulation.
- Develop personalized algorithms for predicting implantation success based on integrated patient data.
- Explore the role of endometrial aging and epigenetics in implantation failures.
- Investigate the molecular cross-talk between embryos and the endometrium to optimize receptivity.
- Conduct large-scale, multi-center trials to validate emerging diagnostic and therapeutic tools.

By advancing these research areas, we can enhance the precision and efficacy of ART, bringing the field closer to the ultimate goal: maximizing the likelihood of a healthy, successful pregnancy for every patient.

## 9 Practical and actionable recommendations for clinicians

Based on the findings of this systematic review and meta-analysis, the following recommendations are provided to guide clinicians in optimizing implantation outcomes and managing patients undergoing assisted reproductive technologies (ART):

### 9.1 Focus on embryo quality

*Prioritize Euploid Embryo Transfer:* Perform PGT-A in eligible patients, particularly those with advanced maternal age or recurrent implantation failure (RIF). The transfer of euploid embryo enhances significantly implantation and live birth rates.

• *Actionable Tip*: Encourage patients to undergo multiple ovarian stimulation cycles, if needed, to increase the chance of obtaining euploid embryos, especially in women aged >37 years.

### 9.2 Optimize endometrial receptivity

*Assess Endometrial Thickness:* Ensure endometrial thickness is >7 mm before transfer, as thin endometrium is associated with lower implantation rates. This issue is still debated and require more robust evidence.

• *Actionable Tip*: Use hormonal therapies such as estrogen supplementation or low-dose aspirin to improve endometrial thickness when suboptimal.

*Diagnose and Treat Chronic Endometritis (CE):* Screen for CE in patients with repeated implantation failure and treat with antibiotics when identified.

• *Actionable Tip*: Perform hysteroscopy or endometrial biopsy for diagnostic clarity in suspected cases of endometrial pathology.

### 9.3 Personalized approaches to timing

*Individualize Embryo Transfer Timing*: Use tools like ERA (Endometrial Receptivity Analysis) or other endometrial differentiation markers (e.g. pinopodes)? or the integration of multiple markers to identify the patient-specific implantation window.

• *Actionable Tip*: Combine endometrial differentiation markers with time-lapse imaging of embryos to match optimal endometrial receptivity with the most viable embryo.

### 9.4 Address age-related challenges

*Proactively Manage Advanced Maternal Age*: Counsel patients about the decline in oocyte quality and endometrial receptivity with age. Offer oocyte donation as a practical option for women with poor ovarian reserve or repeated aneuploid embryos.

• *Actionable Tip*: Set realistic expectations with patients aged >37 years and discuss options like sequential stimulation cycles to optimize outcomes.

### 9.5 Implement immunomodulatory therapies

*Target Immune Dysregulation:* For patients with suspected immune-related implantation failure, consider tailored interventions:

- Use vitamin D supplementation to regulate Th1/Th2 balance.
- Apply tacrolimus or low-dose corticosteroids for immune modulation in select cases.
- Administer low-dose aspirin for thrombophilia or inflammation-related implantation issues.
- *Actionable Tip*: Regularly measure immune markers (e.g., Th1/Th2 ratio, cytokine levels) to assess immune dysregulation and guide treatment.

### 9.6 Optimize gestational carrier programs

*Select Optimal Candidates for GCs:* Use gestational carriers for patients with significant uterine factors or repeated failed transfers despite high-quality embryos.

- *Actionable Tip*: Screen gestational carriers comprehensively, ensuring normal uterine anatomy, endometrial thickness >7 mm (still to be confirmed), and no history of uterine pathology.
- *Manage GC Cycles with PGT-A*: Utilize euploid embryos in GC cycles to maximize implantation and live birth rates.

### 9.7 Establish personalized prediction models

*Leverage Algorithms for Tailored Treatments:* Utilize personalized algorithms incorporating patient data (age, endometrial receptivity, embryo quality) to predict success and guide intervention.

• *Actionable Tip*: Use available ART predictive models and update them with each patient cycle to improve accuracy over time.

### 9.8 Monitor and support lifestyle modifications

*Address Modifiable Risk Factors:* Encourage patients to adopt lifestyle changes that support implantation, including:

Maintaining a healthy BMI.

Reducing stress.

Avoiding smoking and excessive alcohol consumption.

Engaging in regular, moderate physical activity.

• *Actionable Tip*: Work with nutritionists or counsellors to provide tailored support for these lifestyle adjustments.

### 9.9 Set clear patient expectations

*Educate Patients on Success Rates:* Communicate realistic outcomes for ART based on patient-specific factors, such as age and embryo quality.

• *Actionable Tip*: Use data from studies (e.g., 95% success with three euploid embryos) to provide transparent and evidence-based guidance.

### 9.10 Prioritize research-informed practices

*Adopt Evidence-Based Interventions:* Focus clinical efforts on strategies with strong evidence, such as PGT-A, hysteroscopy for uterine abnormalities, and time-lapse imaging.

• *Actionable Tip*: Avoid speculative or unsupported interventions that increase patient costs without proven benefits (e.g., unnecessary immune testing or treatments).

### 9.11 Summary of key actions

- Emphasize euploid embryo transfer with PGT-A.
- Assess and optimize endometrial receptivity using proven methods.
- Offer personalized transfer timing and immune therapies when indicated.
- Use gestational carriers selectively for uterine-factor infertility.
- Integrate patient-specific algorithms for tailored ART protocols.

These practical steps will enable clinicians to apply the findings of this review effectively, ensuring optimal outcomes for patients undergoing ART.

### 9.12 Conclusion

Embryo quality is critical for implantation success, with studies showing a cumulative success rate of over 98% for five sequential euploid embryo transfers (**Tables 5**, **6**). Studies defining embryonic factors often exclude extra-embryonic influences, potentially undervaluing their impact (8–10, 30, 47–82). Evidence indicates this exclusion is flawed, as seen in higher failure rates with single, non-cumulative euploid embryo transfers. Comparisons also show gestational carriers achieve higher implantation rates than transfers into the patient’s own uterus in uterine factor infertility cases (**Figure 5**). Classic research by Csapo and collaborators (83) demonstrated that early pregnancy can be interrupted by luteectomy-induced progesterone withdrawal, mitigated by progesterone replacement until the luteoplacental shift occurs, underscoring hormonal support’s importance. Gestational carriers (GCs) improve ART outcomes by providing a healthy uterine environment, free from barriers that could impact implantation (**Tables 4A**–**D**). The ASRM has set criteria for GCs, including health and pregnancy history, ensuring an optimal environment for embryo development (84). Research focusing solely on embryonic quality risks missing the complexities involved. Controlled trials comparing groups with and without specific extra-embryonic factors, all using euploid embryos, are needed. For RIF, a personalized approach is essential, identifying and addressing potential impediments individually, with the option of a gestational carrier considered where necessary.
